# Flexo-Pyroelectric Effect

**DOI:** 10.34133/research.1048

**Published:** 2026-01-06

**Authors:** Weihao Gao, Shuhai Liu, Yong Qin

**Affiliations:** ^1^ Institute of Nanoscience and Nanotechnology, School of Materials and Energy, Lanzhou University, Lanzhou, Gansu 730000, China.; ^2^ MIIT Key Laboratory of Complex-field Intelligent Exploration, Beijing Institute of Technology, Beijing 100081, China.

## Abstract

Developing sustainable energy technologies is among the foremost challenges of this century and spawns the emergence of materials for ambient energy harvesting, such as photovoltaics, triboelectrics, and pyroelectrics. The conversion of ambient thermal energy (temperature fluctuations) into electricity through pyroelectric materials offers a promising route toward sustainable energy technologies. However, there is a critical limitation: The conventional pyroelectric effect is inherently restricted to noncentrosymmetric crystals, but excludes the centrosymmetric materials, even though they exhibit otherwise favorable properties. This symmetry dependence severely constrains the development of pyroelectric energy technology. Here, we demonstrate that the pyroelectric effect, typically observed only in noncentrosymmetric materials, can be induced in centrosymmetric materials via the flexoelectric effect. By introducing strain gradients using an atomic force microscope, we generated a giant pyroelectric coefficient of up to 1.25 × 10^6^ μC·m^−2^·K^−1^ in SrTiO_3_. This strain gradient-induced pyroelectric effect, termed as the flexo-pyroelectric effect, decouples pyroelectric functionality from intrinsic material polarity. Our findings exceed the long-standing symmetry limitation in pyroelectric energy technology by proving that centrosymmetric materials can also exhibit robust pyroelectricity through strain engineering. The flexo-pyroelectric effect redefines the understanding of pyroelectricity and unlocks the vast library of centrosymmetric materials for designing next-generation energy harvesters, advancing sustainable technology development.

## Introduction

**Fig. 1.**
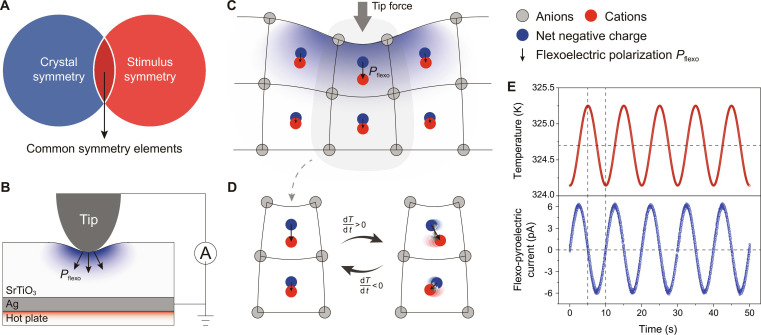
Mechanism of FP effect in centrosymmetric SrTiO_3_ single crystal. (A) Diagram of the principle of crystal symmetry engineering by external stimulus. (B) Setup for temperature fluctuation around the contact between tip and SrTiO_3_ single crystal. The tip loading force is controlled by the feedback loop of a microscale tip indenter or an AFM. (C) Tip loading force-induced inhomogeneous lattice distortion, creating a strong strain gradient (represented by the color gradient and varying lattice spacing), thereby generating the flexoelectric polarization and a local breaking of centrosymmetry. (D) Schematic of the principle of FP effect in centrosymmetric SrTiO_3_ single crystal: temperature fluctuation-induced variation of the flexoelectric polarization. (E) Evolution of the current induced and collected by a conductive microscale tip with a loading force on a SrTiO_3_ (001) face.

Symmetry stands as the basic law of nature that forms the bedrock of modern physics and determines the fundamental physical properties of a material [1], particularly those related to the existence of preferred spatial directions as in a crystal. Breaking the space-inversion symmetry allows emergent functionalities and effects [2]. A notable example is pyroelectricity, which converts temperature fluctuations (temporal temperature gradients *∂T*/*∂t*) into electricity, providing a sustainable energy source to mitigate fossil fuel consumption and carbon emissions [3]. However, this phenomenon is only possible in those materials whose symmetry group lacks an inversion center. Typically, the crystal structure of the material determines the symmetry of the material. Nevertheless, Nye and Lindsay [2] predicted that the material symmetry can also be altered by external stimuli (Fig. 1A) that lowers or even breaks the space-inversion symmetry of any centrosymmetric materials. An exemplary stimulus is the electric field (***E***), which induces electric dipoles and electric polarization (***P*** = *χ**E***, *χ*: dielectric permittivity) in materials, breaking space-inversion symmetry and producing polar structures in centrosymmetric materials [2]. While common in insulators [4], recent observations have also demonstrated pyroelectricity induction by built-in electric field at semiconductor interfaces [5,6]. The practical application of this effect is limited by its requirement for high permittivity and low conductivity [4], This limitation drives the exploration of new approaches for creating and optimizing devices that operate on induced symmetry breaking.

In this regard, the strain gradient (lattice curvature) that creates strain inhomogeneity in materials can play a similar role to the electric field in terms of symmetry engineering [7]. Unlike electric fields, mechanical deformations can penetrate all materials [4], including insulators [8–11], semiconductors [12–14], and metals [15], as “strain cannot be screened”. Inhomogeneous strains can induce electric dipoles in any material [16], underpinning flexoelectricity—the coupling between electric polarization and strain gradients. The latter is caused by asymmetric deformation such as bending and kink, which destroys the equivalence between the 2 sides and leads to asymmetric charge distribution (polarization). Since the lattice asymmetry is due to the deformation itself, any material will undergo flexoelectric polarization regardless of its crystal structure. A wide variety of materials have been reported to exhibit flexoelectricity, including ceramics [8,9], semiconductors [12–14], metals [15], hydrogels [17,18], polymers [19], electrets [20], bones, ear hairs [21], and even frozen water [22]. Essentially, any material that can be bent is able to be polarized.

This symmetry breaking by strain gradient is associated with a variety of emergent functionalities, including piezoelectric [12,23,24], flexomagnetic [15,25], flexoelectronic [13,14,26,27], and bulk photovoltaic effects [28–32], across various materials such as centrosymmetric oxides (SrTiO_3_ and TiO_2_) [12,13,28], halide perovskites (MAPbX_3_, X = Br, Cl, I) [29,32], 2-dimensional materials (MoS_2_ [30] and 3R-MoS_2_ [31]), and more recently ferromagnetic metal (SrRuO_3_ [15]), and even helielectric nematic state (HN*) materials [33]. Likewise, one can be assumed that strain gradients can exhibit pyroelectric effects in materials that are originally centrosymmetric. In this study, we propose and demonstrate that the pyroelectric effect can be induced in any centrosymmetric material via the flexoelectric effect. Given that flexoelectricity is in principle a more general property of all materials, from dielectrics [12] to biomaterials [21], semiconductors [13], and metals [15], to 2-dimensional materials [30,34], this strain gradient-induced pyroelectric effect, here termed as the flexo-pyroelectric (FP) effect, is possible for all symmetry classes. Thus, devices based on the FP effect can be fabricated using materials free from symmetry limitations.

## Results

### Mechanism of the FP effect

To explore our idea, we applied a point force on the surface of centrosymmetric materials (i.e., SrTiO_3_ single crystals, cubic crystal system, space group $\mathrm{Pm}\bar{3}m$) to investigate the induced pyroelectric effect. The point force was exerted by the tip of a custom-made micrometer-scale indentation system (Fig. 1B and Fig. S1), which locally breaks the centrosymmetry via the generated strain gradient (Fig. 1C). In our experiments, the micrometer-scale indentation system was equipped with a femtoampere-level current amplifier-filter and a temperature control system. The temperature control system enabled the precise heating and cooling of sample, achieving an absolute precision of ±0.1 K. A conductive micrometer-scale tip applied a localized force on the sample surface while simultaneously collecting the resultant pyroelectric current.

The SrTiO_3_ single crystal is an ideal platform for studying the flexoelectric effect, owing to their simple cubic centrosymmetric lattices (which excludes piezoelectricity as well as ferroelectricity and facilitates the flexoelectric analysis) and relatively large dielectric permittivity (*χ* > 300 at 300 K, which endows the materials with high flexoelectricity). Unlike its sister material BaTiO_3_, SrTiO_3_ does not exhibit the pyroelectric effect because it has a center of inversion symmetry. However, when the tip loading force breaks the intrinsic centrosymmetry of SrTiO_3_, a flexoelectric polarization is generated, and this induced flexoelectric polarization varies with temperature fluctuation (Fig. 1D). As a result, the temperature fluctuation-induced variation of the flexoelectric polarization theoretically enables the FP effect in the centrosymmetric SrTiO_3_ single crystal. As shown in Fig. 1E, when a sinusoidally varied temperature stimulus is applied around the contact area on a (001) face of an SrTiO_3_ crystal, a marked and stable current that changes in frequency with the temperature is observed under tip force loading. It is worth noting that there is a phase shift of 90° phase difference between the temperature signal and the FP current signal (Fig. 1E). This means that the FP response is not resistive (and thus not a shift current) but rather dielectric in nature, originating from polar displacement. Specifically, if the phase difference between current and temporal temperature gradient is 90°, the polarization (from which the current is the time derivative) is in phase with the temporal temperature gradient.

### Experimental verification of the FP effect

**Fig. 2.**
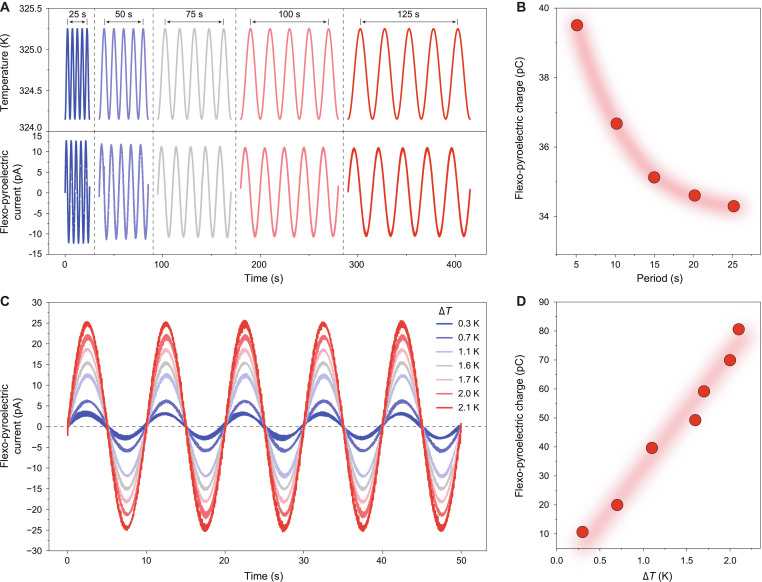
Experimental verification of FP effect. (A) Waveforms of the temperature fluctuation with various periods, along with waveforms of the generated FP current. (B) Temperature fluctuation period dependence of the FP charge. (C) Waveforms of the FP current generated by different amplitude of temperature fluctuation. (D) Temperature fluctuation amplitude dependence of the FP charge.

To solidly demonstrate the validity of the FP effect, we investigated the FP current in response to the temperature fluctuation with controlled period and amplitude. As shown in Fig. 2A, no matter what the period of temperature fluctuation is, the period of FP current is always highly consistent with the period of the temperature, and there is always a phase shift of 90° between them. This consistency of period indicates that the measured signal (FP current) indeed comes from the temperature fluctuation rather than artificial signals nor noise signals. Then, by calculating the integral of the FP current over time, we obtained the corresponding transferred FP charge as a function of the period of temperature fluctuation (Fig. 2B). A clear presence of decreased FP charge with the increased period can be observed. It is easy to understand that as the period becomes larger, the temporal temperature gradient is smaller, resulting in a smaller FP effect. Furthermore, as the amplitude of the temperature fluctuation increases, the FP current also increases accordingly in the same level (Fig. 2C). Through further analysis, we observed a clear linear relationship between the transferred FP charge and the amplitude of temperature fluctuation (Fig. 2D). This linear relationship is consistent with conventional pyroelectric effect, which in turn proves that the measured current indeed originates from the temperature fluctuation and its amplitude highly depends on the temporal temperature gradient.

The FP effect should not be confined to the cubic SrTiO_3_ crystal with cubic crystal. We also performed the same experiment on rutile TiO_2_ single crystals (tetragonal crystal system, space group *P*4_2_/*mnm*) and observed analogous FP current when it is under the action of point force (Fig. S2). This indicates the universal nature of FP effect in centrosymmetric materials. Furthermore, the interface pyroelectric effect previously found in metal–semiconductor heterostructures [5] is also excluded here through material design: adopting undoped SrTiO_3_ and TiO_2_ crystals (considered as insulators) to prevent Schottky barrier formation and build-in field construction [35] at the tip–sample interface, thereby avoiding the possibility of interface pyroelectric effect. Likewise, a potential cubic-to-tetragonal phase transition induced in a SrTiO_3_ single crystal under a large hydrostatic pressure (>6 GPa) should not play a role given the centrosymmetric nature of the induced tetragonal phase [36].

**Fig. 3.**
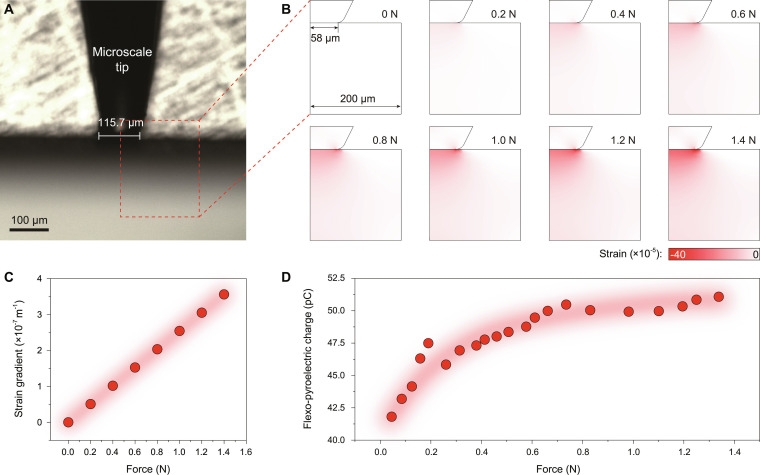
Microscale tip loading force-induced strain gradient and transferred FP charge. (A) Photo image of a microscale tip pressing on the SrTiO_3_ single crystal. (B) Spatial distributions of strain induced by an ideal microscale indenter. (C) Linear relationship between the tip loading force and the force-induced strain gradient. (D) Force dependence of the transferred FP charge.

The strain gradient induced by a microscale tip–sample contact exhibits a complex spatial distribution in an elastic material. The microscale tip apex can be approximated as a cylindrical plateau (Fig. 3A), and the distribution of strain gradient induced by the tip can be calculated analytically with the Hertzian model and the Boussinesq’s calculation [37]. Figure 3B illustrates the spatial distributions of the calculated strain under loading forces of 0 to 1.4 N for a 58-μm-radius contact area, revealing strain gradients linearly proportional to applied force (Fig. 3C). It is expected that the strain gradients induced by the tip loading force will break local symmetry, thus leading to the manifestation of the pyroelectric effect locally under temperature fluctuations. However, deep theoretical analyses are required to understand the intricate relationship between the complex distribution of the strain gradient and the basic behaviors of the FP effect. Experimentally, we found that the transferred FP charge per cycle increases with force up to ~0.7 N, beyond which saturation occurred (Fig. 3D). This saturation is attributed to the mechanical limits of the microscale indentation system. The SrTiO₃ crystal is exceptionally hard, and at high loads, the tungsten steel tip may undergo blunting or plastic deformation. This increases the effective contact area, which in turn reduces the generated strain gradient for a given load. The saturation thus reflects a balance where further force increase is offset by a decreasing strain gradient due to tip deformation, limiting the maximum flexoelectric polarization achievable in this configuration.

### FP effect extended to nanometer scale and its giant magnitude

**Fig. 4.**
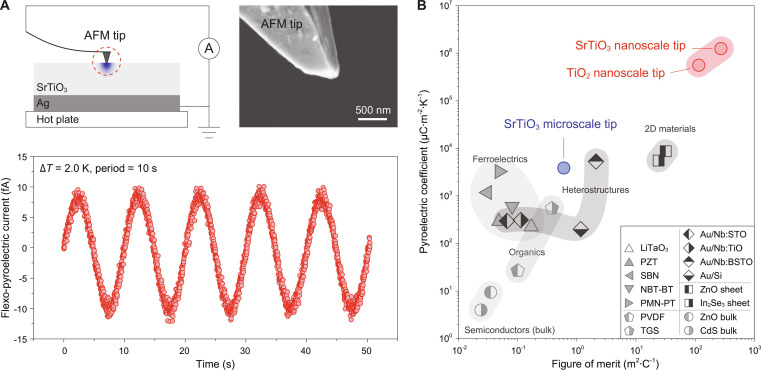
FP effect extended to nanometer scale and its giant magnitude. (A) AFM tip force-induced FP effect. (B) Comparison of pyroelectric coefficients and figures of merit. The FP effect in SrTiO_3_ and TiO_2_, enabled by strain engineering, achieves pyroelectric coefficients that are several orders of magnitude larger than those of best-in-class conventional pyroelectrics (e.g., TGS and PMN-PT) and recently reported interface-based systems (e.g., Au/Nb:BSTO).

The strain gradient induced by tip loading force becomes increasingly significant as tip dimensions decrease to the nanoscale, where the flexoelectric effect is more pronounced [16,28]. This suggests that using a nanometer-scale tip of an atomic force microscope (AFM) rather than a micrometer-scale indentation system may provide a valuable route for strain engineering to enhance the FP effect. To verify this hypothesis, we employed a custom-made pyroelectric atomic force microscope (Pyro-AFM), which integrates an AFM system with a femtoampere-level current amplifier-filter and a temperature-controlled stage (±0.1 K). A conductive AFM tip applied a localized force on the sample surface while simultaneously collecting the resultant FP current. A schematic of the Pyro-AFM setup and a scanning electron microscope (SEM) image of the AFM tip are shown in the upper panel of Fig. 4A. Under a tip loading force (~0.52 μN) and a sinusoidal thermal cycling (*ΔT* = 2.0 K, period = 10 s), a clear sinusoidal FP current synchronized with temperature modulation was observed (Fig. 4A, lower panel). Remarkably, the strain gradient induced by AFM tip yielded a record-breaking pyroelectric coefficient as high as 1.25 × 10^6^ μC·m^–2^·K^–1^.

To confirm that the enhanced FP effect at the nanoscale follows the same fundamental physics as its microscale counterpart, we verified the key scaling relationships. As shown in Figs. S3 to S5, the FP charge maintains a linear dependence on the temperature amplitude Δ*T*, and its magnitude varies inversely with the period of thermal cycling, consistent with a current generation mechanism proportional to the temporal temperature gradient d*T*/d*t*. This confirms that the giant pyroelectric coefficient observed at the nanoscale stems from a massive enhancement of the strain gradient-induced polarization, not a change in the underlying functional dependence on thermal stimuli.

By demonstrating the consistency of these scaling laws across both micro- and nanoscales, we significantly strengthen the argument that the observed phenomenon is the same fundamental FP effect, merely amplified by the enormous strain gradients achievable at the nanoscale. We thank the reviewer for prompting this crucial verification.

### Features of the FP effect

We highlight here 4 main features of the FP effect. First, the pyroelectric coefficient obtained via the FP effect exceeds that of conventional polar materials, even the best ferroelectrics, heterostructures, and 2-dimensional materials (Fig. 4B and Table S1). For example, the pyroelectric coefficient of SrTiO_3_ achieved through the FP effect is over 6.9 times larger than that of triglycine sulfate (TGS) and 19 times larger than that of Au/Si, both of which have similar figures of merit. Beyond oxide semiconductors such as SrTiO_3_ and TiO_2_, there is great potential to enhance the pyroelectric coefficient by exploring materials with a large flexoelectric effect, such as paraelectric phases of polycrystalline ferroelectrics wherein the flexoelectric coefficient is over 3 orders of magnitude larger than that of SrTiO_3_ crystal. Second, a high pyroelectric current can be obtained from any material by simply generating a large strain gradient. For instance, a sharp probe with sufficient loading force can be used for bulk materials, or microstructures with sharp contours can be employed for 2-dimensional materials. Third, unlike the conventional pyroelectric effect that is limited to noncentrosymmetric materials, the FP effect is universal. It is allowed in all materials with any symmetry due to the universal nature of strain gradient-induced centrosymmetry breaking. The FP effect not only is observed in SrTiO_3_ and TiO_2_ but also is applicable to any flexoelectric material, ranging from ferroelectric ceramics [8,9], halide perovskites (MAPbX_3_, X = Br, Cl, I) [29,32], 2-dimensional materials (MoS_2_ [30] and 3R-MoS_2_ [31]), ferromagnetic metal (SrRuO_3_ [15]), polymers [19], electrets [20], biological materials such as bones and ear hairs [21], and even frozen water [22] and helielectric nematic state (HN*) materials [33]. Fourth, the FP effect exhibits a strong size effect. As the tip size decreases from the micrometer-scale to the nanometer-scale, the pyroelectric coefficient can even increase by 326 times. This size effect is highly similar to that of flexoelectric effect, which in turn can highlight the important role of the tip loading force-induced flexoelectric polarization in the FP effect. Meanwhile, this huge improvement further indicates that the FP effect plays a role, rather than the previously reported interface pyroelectric effect [5] or the surface pyroelectric effect [38]: If the current is generated by the interface pyroelectric effect or surface pyroelectric effect, it will not increase gigantically as tip dimensions decrease to the nanoscale.

To further demonstrate the universality of the FP effect beyond oxide semiconductors, we investigated a polyvinylidene fluoride (PVDF) polymer film and a silicon wafer, representing a flexible organic system and an elemental semiconductor, respectively. As shown in Fig. S6, clear and phase-locked FP currents were observed under AFM tip loading in both materials. This conclusively shows that the FP effect is a general phenomenon, applicable across a vast range of material classes, from rigid oxides to soft polymers and elemental semiconductors, thereby dramatically expanding the library of candidate materials for pyroelectric applications. Furthermore, our pyroelectric indentation system is straightforward to configure, and the FP effect grows significantly at the nanoscale, a region where the flexoelectric effect is most dominant [16]. This establishes a compelling strain engineering strategy for improving the performance of pyroelectric devices. For example, an integrated pyroelectric harvester can be fabricated by combining an array of nanoscale indenters (e.g., nanowire arrays) [39] with 2-dimensional materials (e.g., single-layer MoS_2_ and nanomembranes) [40], thereby enabling a higher efficiency because the FP effect can be designed to increase the existing pyroelectric current in the polar materials. Considering the considerable strain gradients produced by interface lattice mismatch and crystallographic disorder in polycrystalline films [30,31,41], the associated FP effect could significantly enhance the performance of these pyroelectric harvesters, although this area remains largely unexplored. In addition to inorganic pyroelectric energy harvesters, the FP effect is also poised to be key for flexible and stretchable electronics that use organic and polymer materials. Both macroscopic bending of flexible organic devices [20] and nanoscale folding and entanglement of polymeric chains generate sizable strain gradients [41,42], which can modify electronic transport and induce the FP effect under temperature fluctuations; however, the details of how strain gradients and the FP effect influence electronic characteristics of nanoscale systems remain a topic of ongoing debate.

## Discussion

In summary, we have found the FP effect that not only exhibits substantial pyroelectric coefficients but also overcomes the symmetry limitations of conventional pyroelectric materials. The FP effect is applicable to a wide range of materials, from conventional semiconductors and oxides to halide perovskites and 2-dimensional materials. These features enable their practical applications in thermal effect-related fields, such as energy conversion and infrared sensing, with distinctive mechanisms and additional tuning capabilities distinct from those of intrinsic noncentrosymmetric materials.

## Materials and Methods

### Experiment setup and characterization for the FP current

SrTiO_3_ and TiO_2_ samples were ultrasonically cleaned in ethanol and acetone and then bonded to a conductive substrate using silver paste. The substrate assembly was placed on a DC-powered thermal stage. The temperature of the substrate was precisely controlled by supplying DC power to the thermal stage using a Keithley 2636A Source Meter. A conductive tungsten steel microprobe was vertically pressed onto the surface of the SrTiO_3_ sample. The applied pressure was monitored and measured using a force gauge (DS2-2N). For pyroelectric signal measurement, the conductive substrate was connected to the cathode and the probe was connected to the anode of a Keithley B2985A Electrometer/High Resistance Meter. The FP current signals were directly measured using the Keithley B2985A. The basic properties of SrTiO_3_ and TiO_2_ crystals are listed in Table S2.

### Pyro-AFM setup for pyroelectric current measurement

The sample was similarly bonded to a conductive substrate using silver paste. Temperature control was maintained via the DC-powered thermal stage positioned beneath the substrate. The AFM (Bluker Dimension Icon) setup was operated in contact mode. A specialized C-AFM Probe Holder, featuring an integrated wire lead at its rear for electrical connection, was employed. The conductive AFM tip was stably engaged onto the sample surface under constant force (0.52 μN). For electrical measurement, the integrated wire lead of the C-AFM Probe Holder was connected to the anode of the Keysight B2985A, while the conductive substrate was connected to the cathode. The Keysight B2985A instrument was used to directly measure the temperature-dependent pyroelectric current flowing through the sample and the AFM tip.

The FP current signals were directly measured using a Keysight B2985A Electrometer/High Resistance Meter. To ensure optimal stability and accuracy for the pA-level and fA-level currents, the instrument was operated in the pA or fA fixed range mode (as opposed to auto-range) to prevent range-switching artifacts during measurement. The integration time was set to the “Short” power line cycle (PLC) setting to provide a suitable balance between measurement noise and temporal resolution for the observed current signals. All measurements were conducted in a shielded probe station to minimize electromagnetic interference.

## Data Availability

The datasets used and analyzed during the current study are available from the corresponding authors on reasonable request.

## Supplementary Materials

Supplementary 1 Figs. S1 to S6 Tables S1 and S2 References [43–50]
