# Supplementary material for: Flexo-Pyroelectric Effect
Source: Research (Wash D C). 2026 Jan 6;9:1048. doi: 10.34133/research.1048 (PMC12770234; doi:10.34133/research.1048)
Supplement: Supplementary 1 — Figs. S1 to S6 Tables S1 and S2 References [43–50] [file research.1048.f1.docx]

Supplementary Materials for

**Flexo-Pyroelectric Effect**

Weihao Gao^1,2^, Shuhai Liu^1,2^*, Yong Qin^1,2^*

^1^Institute of Nanoscience and Nanotechnology, School of Materials and Energy, Lanzhou University, Lanzhou, Gansu 730000, China.

^2^MIIT Key Laboratory of Complex-field Intelligent Exploration, Beijing Institute of Technology, Beijing 100081, China.

*Address correspondence to: liushuhai1991@live.cn, qinyong@lzu.edu.cn.

**This PDF file includes:**

Figures S1 to S6

Tables S1 to S2


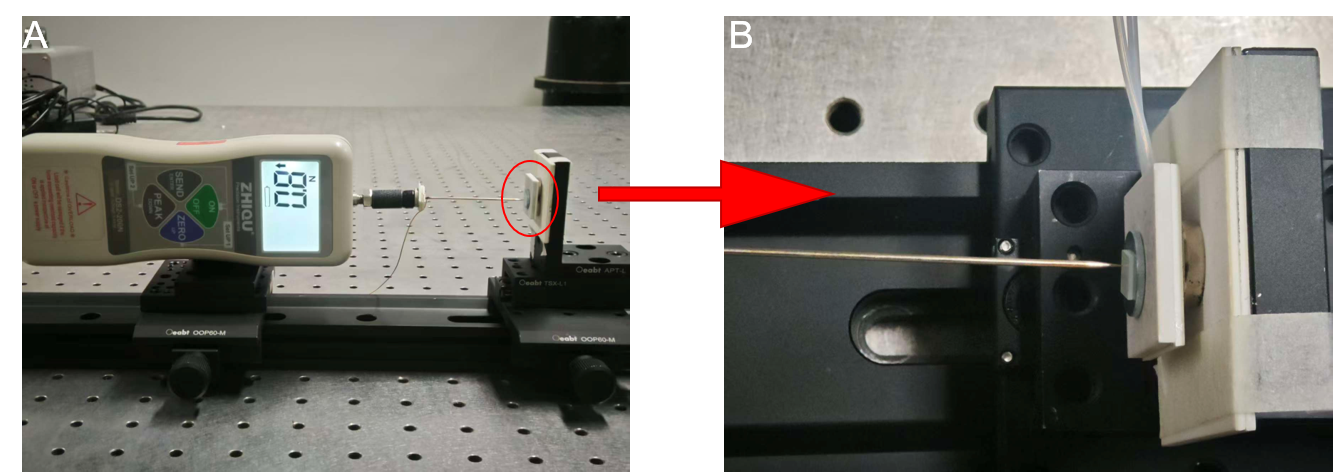


**Fig. S1.** **Photographs of** (**A**) **experimental device and** (**B**) **partial details.**


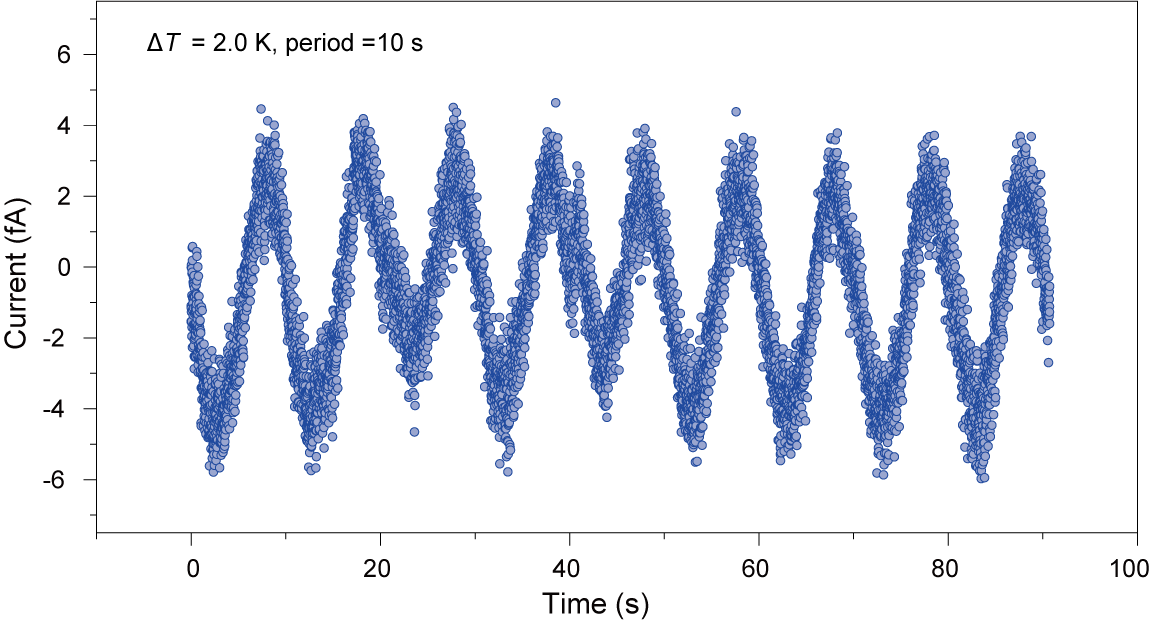


**Fig. S2.** **FP effect in TiO_2_ single crystal.**


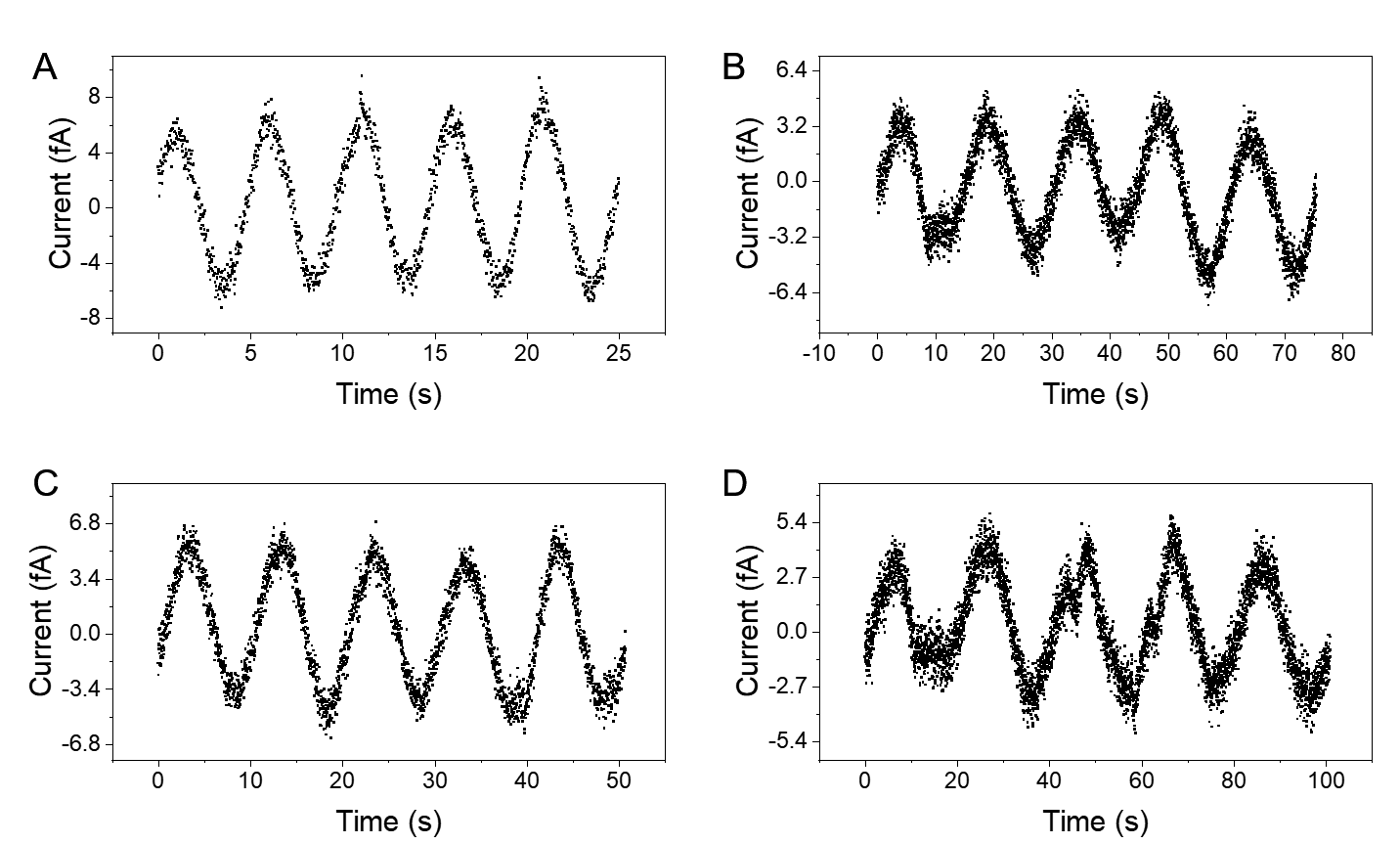


**Fig. S3. Pyroelectric current signals with different cycle times.**


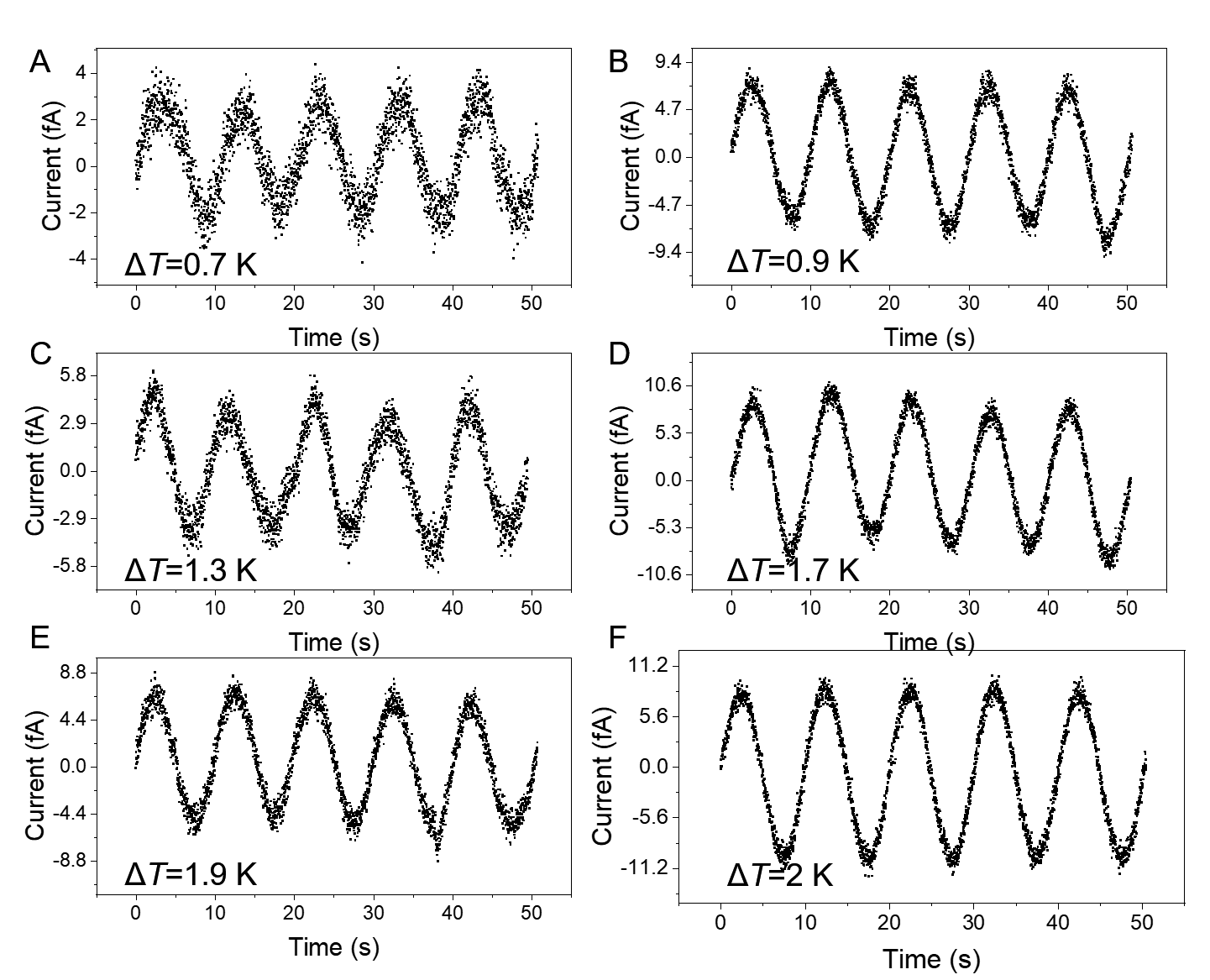


**Fig. S4. Pyroelectric current signal under different temperature difference.**


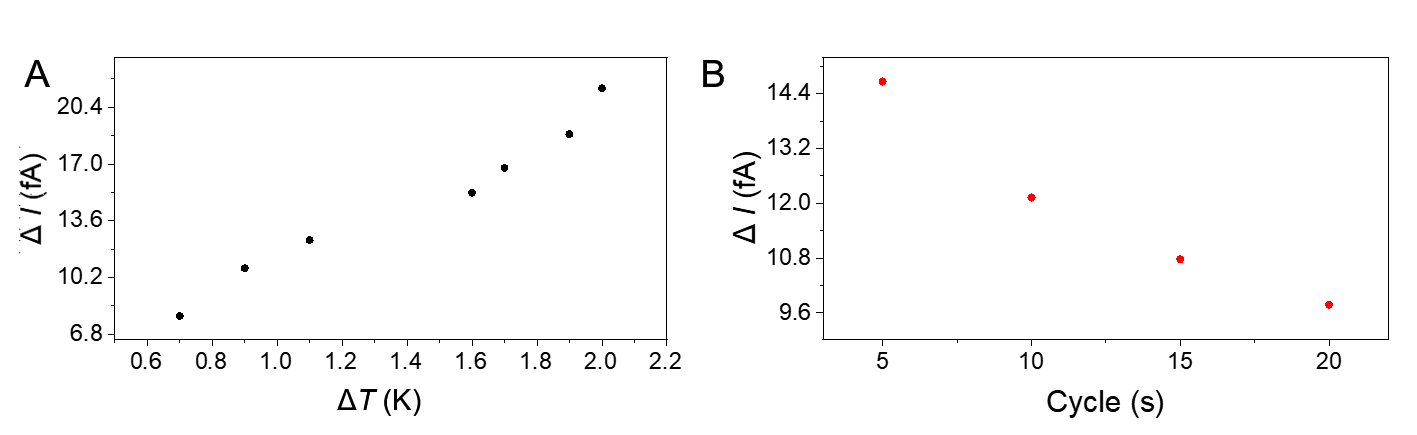


**Fig. S5.** (**A**) Temperature fluctuation amplitude dependence of the FP charge. (**B**) Temperature fluctuation period dependence of the FP charge.


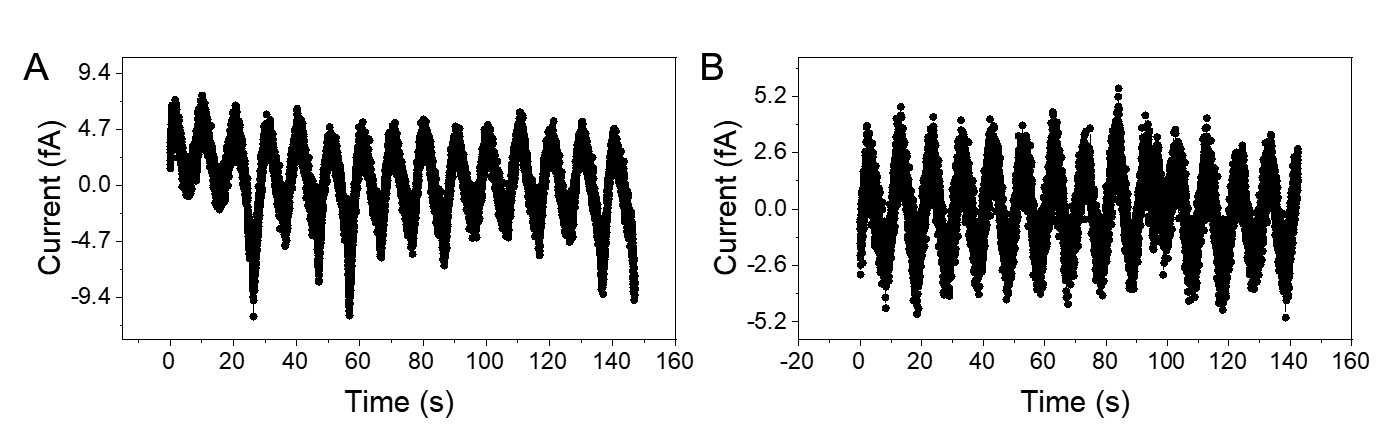


**Fig. S6.** **The FP signal of (A) PVDF film and (B) silicon wafer.**

**Table S1.** **Summary of figure of merit and pyroelectric coefficient of typical pyroelectric materials.**

| Materials | Figure of merit  (m^2^ C^–1^) | Pyroelectric coefficient  (μC m^2^ K^–1^) | References |
| --- | --- | --- | --- |
| SrTiO_3_ AFM tip | 275.57 | 1.25×10^6^ | This work |
| TiO_2_ AFM tip | 115.72922 | 5.58×10^5^ | This work |
| SrTiO_3_ probe | 0.61 | 3830 | This work |
| CdS bulk | 0.024 | 4 | (*43*) |
| ZnO bulk | 0.035 | 9.4 | (*44*) |
| PMN-PT | 0.05 | 3260 | (*45*) |
| Pb(Ti_0.8_Zr_0.2_)O_3_  (PZT) film | 0.0481 | 300 | (*46*) |
| Sr_0.5_ Ba_0.5_Nb_2_O_6_  (SBN) | 0.03 | 1180 | (*47*) |
| Mn:0.946Na_0.5_Bi_0.5_TiO_3_-0.054BaTiO_3_  (NBT-BT) | 0.08 | 588 | (*48*) |
| polyvinylidene fluoride (PVDF) | 0.1 | 27 | (*49*) |
| triglycine sulfate  (TGS) | 0.38 | 550 | (50) |
| LiTaO_3_ | 0.17 | 230 | (*49*) |
| Au/Nb:SrTiO_3_  (Au/Nb:STO) | 0.066 | 298 | (*5*) |
| Au/Nb:TiO_2_  (Au/Nb:TiO) | 0.113 | 312 | (*5*) |
| Au/Nb:Ba_0.6_Sr_0.4_TiO_3_  (Au/Nb:BSTO) | 2.11 | 5300 | (*5*) |
| Au/Si | 1.167 | 200 | (*5*) |
| In_2_Se_3_ sheet | 24.2 | 5526.2 | (*40*) |
| ZnO sheet | 31.9 | 8683.7 | (*40*) |

Note: The values listed are from the cited references and were obtained under various measurement conditions (e.g., frequency, temperature range). The table is intended to provide a general performance comparison, highlighting the significant enhancement achieved via the flexo-pyroelectric effect. For precise performance evaluation of a specific material, the reader is referred to the original publications.

**Table S2.** **Properties of** **SrTiO_3_ and TiO_2_ crystals.**

| Categories | SrTiO_3_ | TiO_2_ |
| --- | --- | --- |
| Crystal structure | Cubic, *a* = 3.905 Å | Tetragonal, *a* = 3.905 Å, *c* = 2.9582 Å |
| Growth method | Vernuil | Floating zone method |
| Roughness of surface | Within 5 Å | Within 5 Å |
| Density | 5.175 g·cm^–3^ | 4.26 g·cm^–3^ |
| Hardness | 6 (Mohn) | 6.5 (Mohn) |
| Dielectric permittivity | 1.68 × 10^–9^ C·V^–1^·m^–1^ | 1.68 × 10^–9^ C·V^–1^·m^–1^ |
| Relative dielectric constant | 190 | 190 |
| Specific heat capacity | 2.7 J K^–1^ cm^–3^ | 2.87 J K^–1^ cm^–3^ |
